# Association between hyperlipidemia and postoperative delirium risk: a systematic review and meta-analysis

**DOI:** 10.3389/fnagi.2025.1544838

**Published:** 2025-03-18

**Authors:** Li-quan Qiu, Jian-li Song, Li-cai Zhang, Bin Fan, Qiang Li, Bin Lu, Guan-yu Chen

**Affiliations:** Department of Anesthesiology, Zigong Fourth People's Hospital, Zigong, Sichuan, China

**Keywords:** meta-analysis, postoperative delirium, hyperlipidemias, hypertriglyceridemia, hypercholesterolemia

## Abstract

**Introduction:**

The association between hyperlipidemia and its potential role as a risk factor for postoperative delirium (POD) remains unclear.

**Methods:**

We systematically searched PubMed, Embase, Web of Science, Cochrane Library, and ClinicalTrials.gov to identify studies meeting the inclusion criteria. Outcomes of interest included comparisons of blood lipid levels between POD and non-POD (NPOD) patients, the association between hyperlipidemia and POD risk, and the predictive value of hyperlipidemia for POD.

**Results:**

A total of nine studies involving 4,686 patients were included in the meta-analysis. Pooled analysis revealed that hyperlipidemia was significantly associated with an increased risk of POD (OR = 1.47; 95% CI 1.13–1.91; *P* = 0.004;) compared to patients without hyperlipidemia. Patients with POD exhibited significantly higher levels of total cholesterol (TC) (weighted mean difference [WMD] = 0.31; 95% CI 0.03–0.59; *P* = 0.030), triglycerides (TG) (WMD = 0.37; 95% CI 0.03–0.71; *P* = 0.033), and low-density lipoprotein cholesterol (LDL-C) (WMD = 0.09; 95% CI 0.01–0.17; *P* = 0.023) compared to NPOD patients. In contrast, high-density lipoprotein cholesterol (HDL-C) levels were significantly lower in POD patients (WMD = −0.07; 95% CI −0.12 to −0.01; *P* = 0.026). Insufficient evidence was available to summarize the area under the curve (AUC) results.

**Conclusions:**

Blood lipid levels were significantly elevated in POD patients compared to NPOD patients. Hyperlipidemia was significantly associated with an increased risk of POD, highlighting its potential role as a risk factor.

## Introduction

Postoperative delirium (POD) is an acute and reversible form of brain dysfunction characterized by inattention, altered consciousness, and sudden cognitive decline. It is a common complication following anesthesia and surgery, with incidence rates ranging from 50 to 70% in high-risk populations (Swarbrick and Partridge, 2022; Wang et al., 2020; Jin et al., 2020). POD can prolong hospital stays, increase healthcare costs, raise postoperative mortality, and elevate the risk of other complications, thereby negatively impacting patient outcomes (Inouye et al., 2014; Bai et al., 2020; Yan et al., 2023). The etiology of POD is multifactorial, encompassing a range of risk factors such as advanced age, pre-existing cognitive impairment, chronic alcoholism, the presence of comorbid conditions, and variations related to specific surgical procedures (Gravante et al., 2021; Kazmierski et al., 2022). Currently, there is no specific treatment for POD. Therefore, it is essential to investigate perioperative risk factors and implement proactive interventions to reduce the incidence and severity of POD, ultimately improving postoperative recovery.

Hyperlipidemia, also known as dyslipidemia, is a metabolic disorder characterized by abnormal lipid levels, typically presenting as elevated total cholesterol (TC), triglycerides (TG), and low-density lipoprotein (LDL), or reduced high-density lipoprotein (HDL) levels (Li et al., 2023). This condition can lead to lipid deposition in vascular endothelium, contributing to atherosclerosis, blood-brain barrier disruption, and abnormal lipid accumulation in the brain, potentially resulting in neurodegenerative diseases. Dysregulated lipid metabolism is strongly associated with Alzheimer's disease, with significant differences in blood lipid levels observed between cognitively impaired patients and healthy individuals (Buckley et al., 2018; Pérez-Gálvez et al., 2018). Given the shared pathophysiological mechanisms between Alzheimer's disease and POD, it is plausible that abnormal lipid metabolism may also increase the risk of POD, a hypothesis supported by recent observational studies (Fong et al., 2021). Specifically, hyperlipidemia has been linked to a higher incidence of POD in patients undergoing hip replacement and colorectal cancer surgeries (Zhao et al., 2024; Lin et al., 2022); however, some studies suggest that POD may be associated only with low HDL levels rather than triglyceride levels (Feinkohl et al., 2023). Therefore, the relationship between hyperlipidemia and POD remains controversial.

Several meta-analyses have been published investigating risk factors for POD in various surgical cohorts, including gastrointestinal and orthopedic surgeries (Scholz et al., 2016; Rong et al., 2021). Although these studies have examined hyperlipidemia as one of the potential risk factors, their primary focus was not specifically on hyperlipidemia, and they did not resolve the ongoing controversy regarding the relationship between hyperlipidemia and POD. The research design limited the depth and breadth of the investigation into hyperlipidemia (Scholz et al., 2016; Rong et al., 2021). For example, hyperlipidemia was only analyzed as one of many potential factors, without a thorough exploration of the specific associations between different types, severity levels, and other characteristics of hyperlipidemia and POD. To address this gap, we conducted the first systematic review and meta-analysis to evaluate the association between hyperlipidemia and the incidence of POD.

## Methods

This study was performed according to the Preferred Reporting Items for Systematic Reviews and Meta-Analyses (PRISMA) statement. The protocol for this meta-analysis is available in PROSPERO (CRD42024588137) (Shamseer et al., 2015).

### Search for trials

We conducted a comprehensive search of the PubMed (from 1966), Web of Science (Science Citation Index Expanded, from 1900), Cochrane Library (from 1993), ClinicalTrials.gov (from 2000), and Embase (from 1947) databases, and the search spanned from these starting dates through September 9, 2024. The search utilized the following keywords: “postoperative delirium,” “hyperlipidemias,” “hypertriglyceridemia,” “hypercholesterolemia,” and “delirium,” aiming to identify studies that met the predefined inclusion criteria. Furthermore, references from all eligible articles were meticulously examined to identify additional potentially relevant studies. Any discrepancies were resolved through discussion with a third author (CGY). The detailed search strategy for PubMed is provided in Supplementary File 1.

### Selection criteria and inclusion criteria

The inclusion criteria were as follows: (1) Study population: adult patients scheduled for surgery; (2) Study design: observational studies (cohort studies or case-control studies); (3) Exposure: hyperlipidemia as the exposure factor, with patients categorized into hyperlipidemia and non-hyperlipidemia groups; (4) Outcome: comparison of blood lipid levels between POD and NPOD group—where the NPOD group consisted of patients who underwent the same surgical procedures as the POD group but did not meet the diagnostic criteria for POD—along with an evaluation of the association between hyperlipidemia and POD risk, and an assessment of the predictive value of hyperlipidemia for POD; (5) Data availability: studies providing odds ratios (ORs) and area under the curve (AUC) data; and (6) Language: studies published in English.

Exclusion criteria: studies with incomplete or unavailable data were excluded. Additionally, reviews, case reports, and animal studies were not considered.

### Data extraction and key measurements

The extracted data included study characteristics such as the first author, year of publication, country, study design, sample size, definition of hyperlipidemia, method of POD evaluation, the variables used in the multivariate model, and the number of POD cases. The outcomes of interest encompassed: (1) comparison of blood lipid levels between POD and non-POD (NPOD) groups, (2) the association between hyperlipidemia and POD risk, and (3) the predictive value of hyperlipidemia for POD. Additionally, odds ratios (ORs) and area under the curve (AUC) values were extracted. For ORs, multivariate-adjusted ORs were prioritized for extraction and analysis. If multivariate-adjusted values were unavailable, univariate-adjusted ORs were used instead. Any discrepancies in data extraction were resolved through consensus.

### Quality of evidence

The quality of evidence for all studies was evaluated independently by two authors (JLS and LQQ) utilizing the Newcastle-Ottawa Scale (NOS) criteria applicable to cohort and case-control studies (Stang, 2010). The Newcastle-Ottawa Scale (NOS) assesses the risk of bias across three domains: participant selection, comparability, and outcomes. In the selection and outcome categories, each item is scored with a maximum of 1 point, while the comparability domain allows for a maximum of 2 points. The total score on the NOS ranges from 0 to 9, with higher scores indicating better study quality. Based on the total score, studies were categorized into three quality levels: low (0–3 points), moderate (4–6 points), and high (7–9 points). Any discrepancies in ratings were resolved by a third author (CGY).

### Statistical analysis

We conducted the meta-analysis using STATA software version 14.0 (StataCorp, College Station, TX). Weighted mean differences (WMDs), odds ratios (ORs), area under the curve (AUC) values, and their corresponding 95% confidence intervals (CIs) were used to evaluate the outcomes. Heterogeneity was assessed using the *I*^2^ statistic, and pooled analyses were performed using a random-effects model when significant heterogeneity was observed (*I*^2^ ≥ 50%); otherwise, a fixed-effects model was applied. To address potential statistical or clinical heterogeneity, subgroup analyses were conducted based on country, delirium assessment scale, type of surgery (cardiac vs. non-cardiac), region (Asia vs. Europe), variables adjusted (multivariate vs. univariate) and study design (cohort study vs. case-control). Sensitivity analyses were performed to assess the robustness and quality of the findings by sequentially excluding individual studies from the pooled analyses. Sensitivity analyses were conducted to evaluate the robustness and reliability of the findings by sequentially excluding individual studies from the pooled analyses. Additionally, sensitivity analyses were performed by excluding studies that reported only unadjusted odds ratios (ORs). Publication bias was evaluated using Begg's and Egger's tests, complemented by funnel plots. A *p*-value of < 0.05 was considered statistically significant for all analyses.

## Results

### Studies retrieved and their characteristics

**Figure 1:**
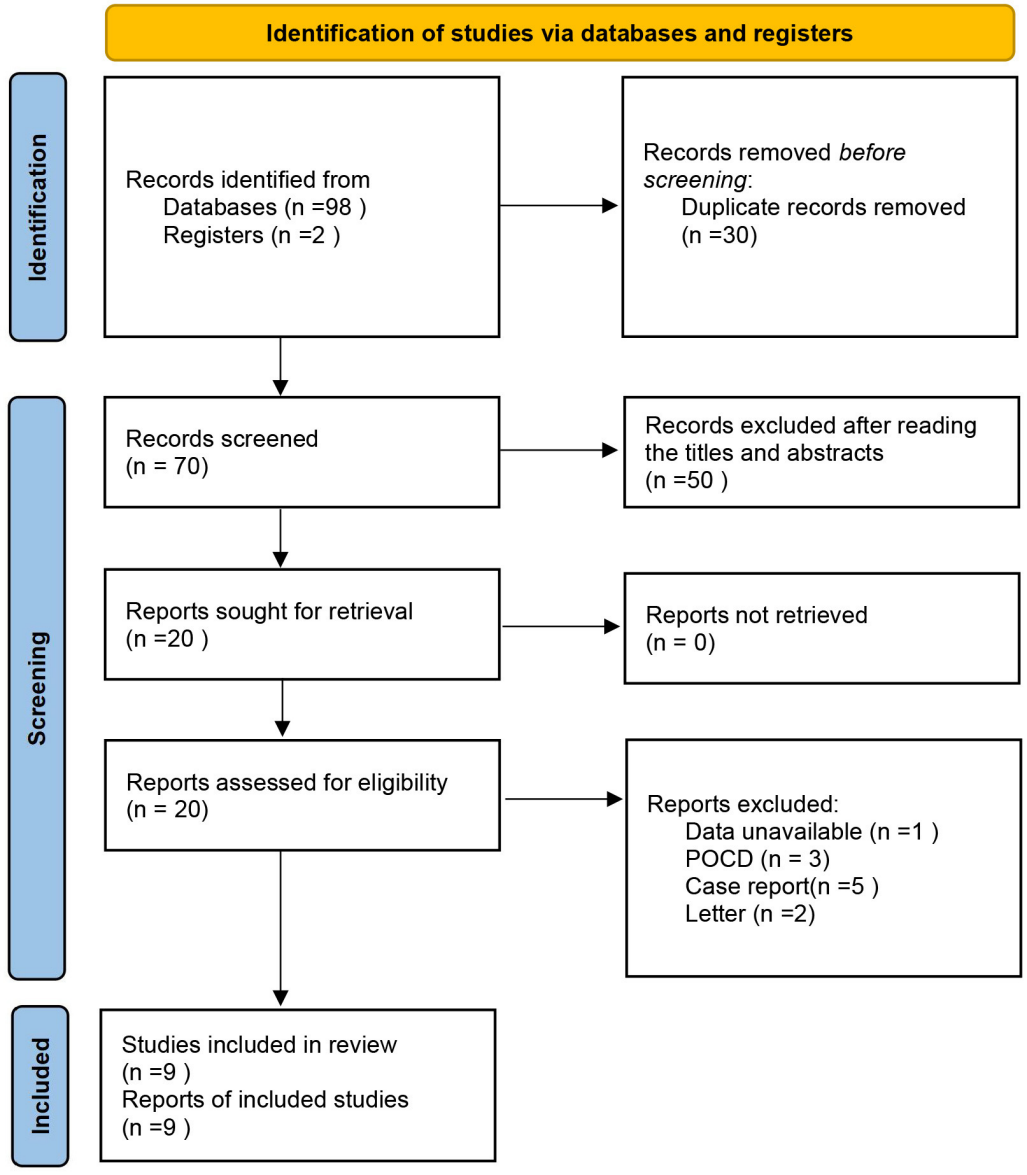
Flow chart for study selection. PRISMA 2020 flow diagram for new systematic reviews which included searches of databases and registers only.

The database search identified 98 potentially eligible records. After screening the titles and abstracts, 20 full-text articles were reviewed, of which 9 met the inclusion criteria (Feinkohl et al., 2023; Lin et al., 2022; Zhao et al., 2024; Böhner et al., 2003; Chu et al., 2021; Ding et al., 2024; Li et al., 2021; Sugimoto et al., 2015; Wang et al., 2015) (Figure 1).

The characteristics of the included studies are summarized in Supplementary Table 1. The sample sizes ranged from 130 to 1,462 participants, with a total of 4,686 individuals included in the meta-analysis. Among these, 1,104 (23.6%) developed postoperative delirium (POD), while 3,582 (76.4%) did not. Geographically, seven studies (77.8%) were conducted in Asia—including six from China and one from Japan—and two (22.2%) were from Europe, both of which were conducted in Germany.

For delirium assessment, two studies used the Confusion Assessment Method for the Intensive Care Unit (CAM-ICU), three employed the standard Confusion Assessment Method (CAM), two applied the Diagnostic and Statistical Manual (DSM) criteria, one utilized the 3-min Diagnostic Interview for Confusion Assessment Method (3D-CAM), and one study incorporated multiple assessment scales.

In terms of study design, three were retrospective case-control studies, and six were prospective cohort studies. Regarding surgical procedures, two studies focused on cardiac surgeries, while the remaining seven involved non-cardiac surgeries. For hyperlipidemia definitions, only four studies provided specific criteria, whereas the other five used a generalized definition without distinguishing between low-density lipoprotein cholesterol (LDL-C), high-density lipoprotein cholesterol (HDL-C), total cholesterol (TC), and triglyceride (TG) levels.

As for anesthesia techniques, five studies used general anesthesia, one employed an epidural block, one combined general anesthesia with an epidural block, and two did not specify the anesthesia methods. Among the included studies, four adjusted for multiple confounding factors and reported adjusted odds ratios (ORs), while the remaining five studies provided unadjusted ORs without covariate adjustment.

### Quality of evidence

The NOS overall methodological score of all included studies revealed moderate to high quality (Supplementary Tables 2, 3).

### Comparison of blood lipid levels between POD and NPOD

**Figure 2:**
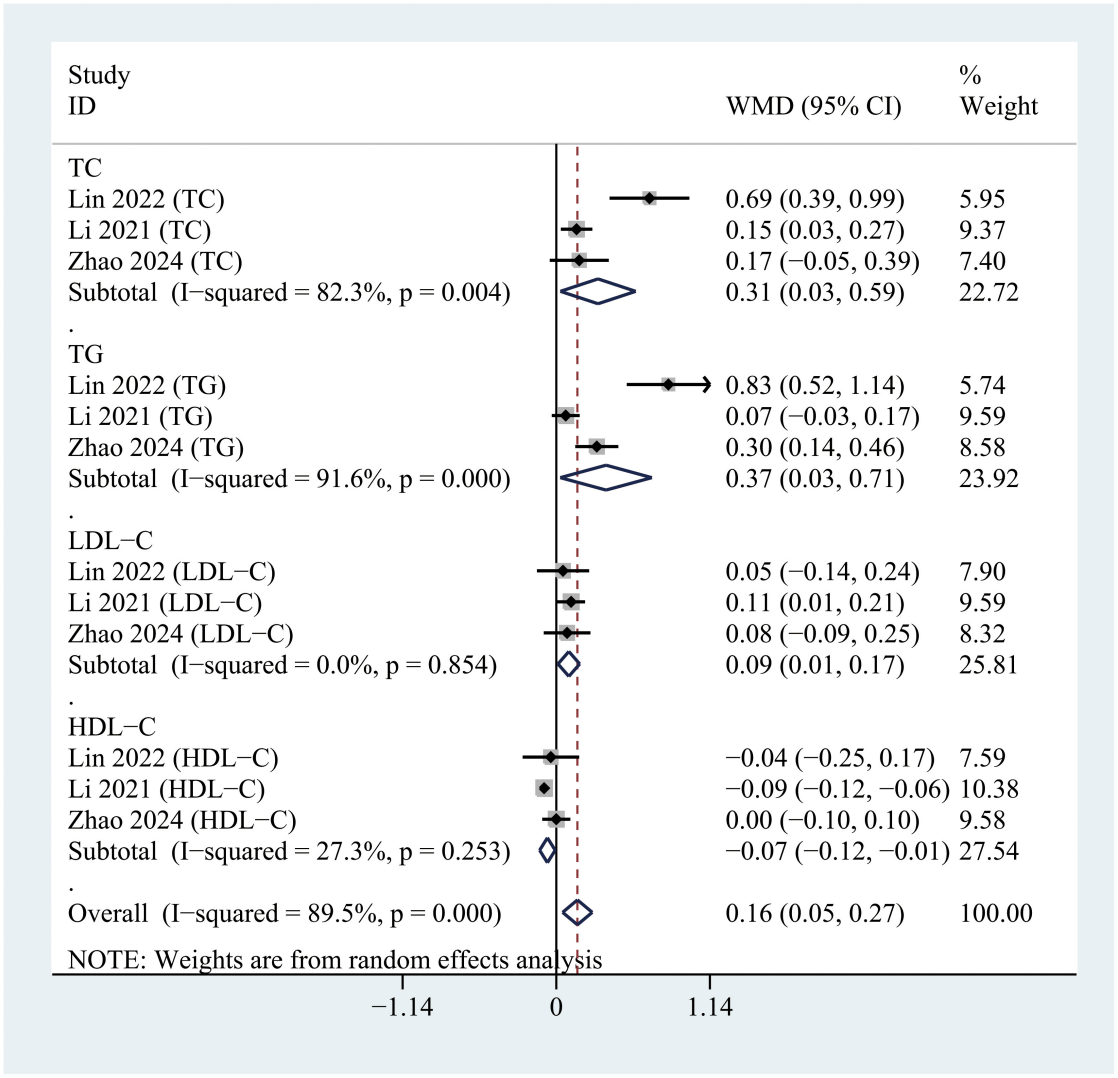
Forest plot comparing serum lipid profiles, including total cholesterol (TC), triglycerides (TG), low-density lipoprotein cholesterol (LDL-C), and high-density lipoprotein cholesterol (HDL-C), between patients with and without postoperative delirium.

The pooled results demonstrated that patients with postoperative delirium (POD) had significantly higher levels of TC (WMD = 0.31; 95% CI 0.03–0.59; *P* = 0.030; I^2^= 82.3%), TG (WMD = 0.37; 95% CI 0.03–0.71; *P* = 0.033; I^2^= 91.6%), and LDL-C (WMD = 0.09; 95% CI 0.01–0.17; *P* = 0.023; I^2^= 0%) compared to non-POD (NPOD) patients. In contrast, HDL-C levels were significantly lower in POD patients (WMD = −0.07; 95% CI −0.12 to −0.01; *P* = 0.026; I^2^= 27.3%) relative to NPOD patients (Figure 2).

### Association between hyperlipidemia and risk of POD

**Figure 3:**
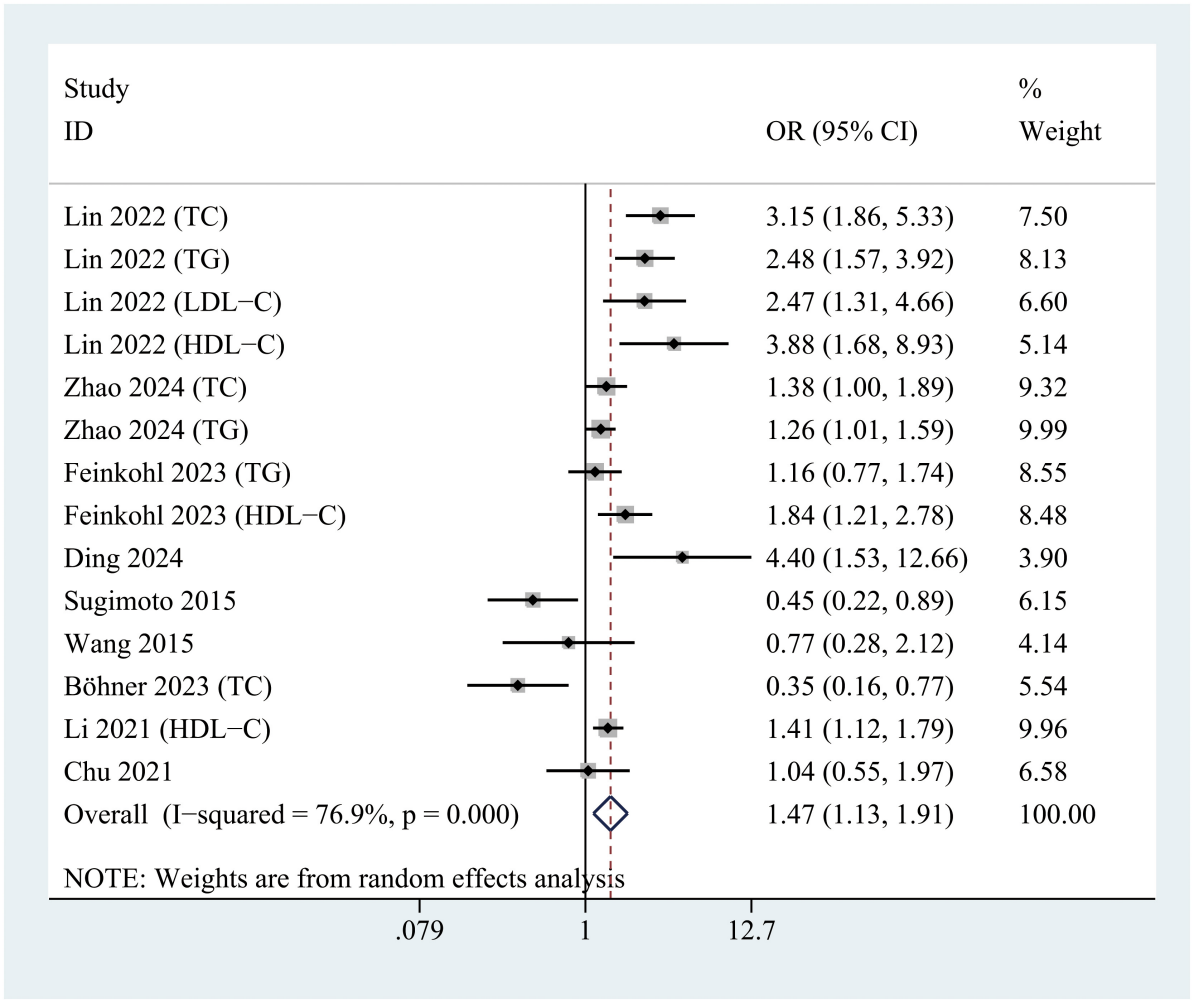
Forest plot demonstrating the association between hyperlipidemia and the risk of postoperative delirium development.

All included studies were analyzed to evaluate the association between hyperlipidemia and the risk of POD. The pooled analysis revealed that hyperlipidemia was significantly associated with an increased risk of POD (OR = 1.47; 95% CI 1.13–1.91; *P* = 0.004; I^2^= 76.9%) compared to patients without hyperlipidemia (Figure 3).

### Predictive value of hyperlipidemia for POD

Only one study included in the meta-analysis reported area under the curve (AUC) results, which precluded a comprehensive summary analysis. However, the receiver operating characteristic (ROC) curves indicated that LDL-C [AUC = 0.607 (0.519–0.691)], HDL-C [AUC = 0.620 (0.531–0.703)], TG [AUC = 0.761 (0.679–0.831)], and TC [AUC = 0.708 (0.623–0.784)] were all predictive of POD.

### Subgroup analyses

Subgroup analyses were conducted based on country, delirium assessment scale, type of surgery (cardiac vs. non-cardiac), region (Asia vs. Europe), variables adjusted (multivariate or univariate) and study design (cohort study vs. case-control).

**Figure 4:**
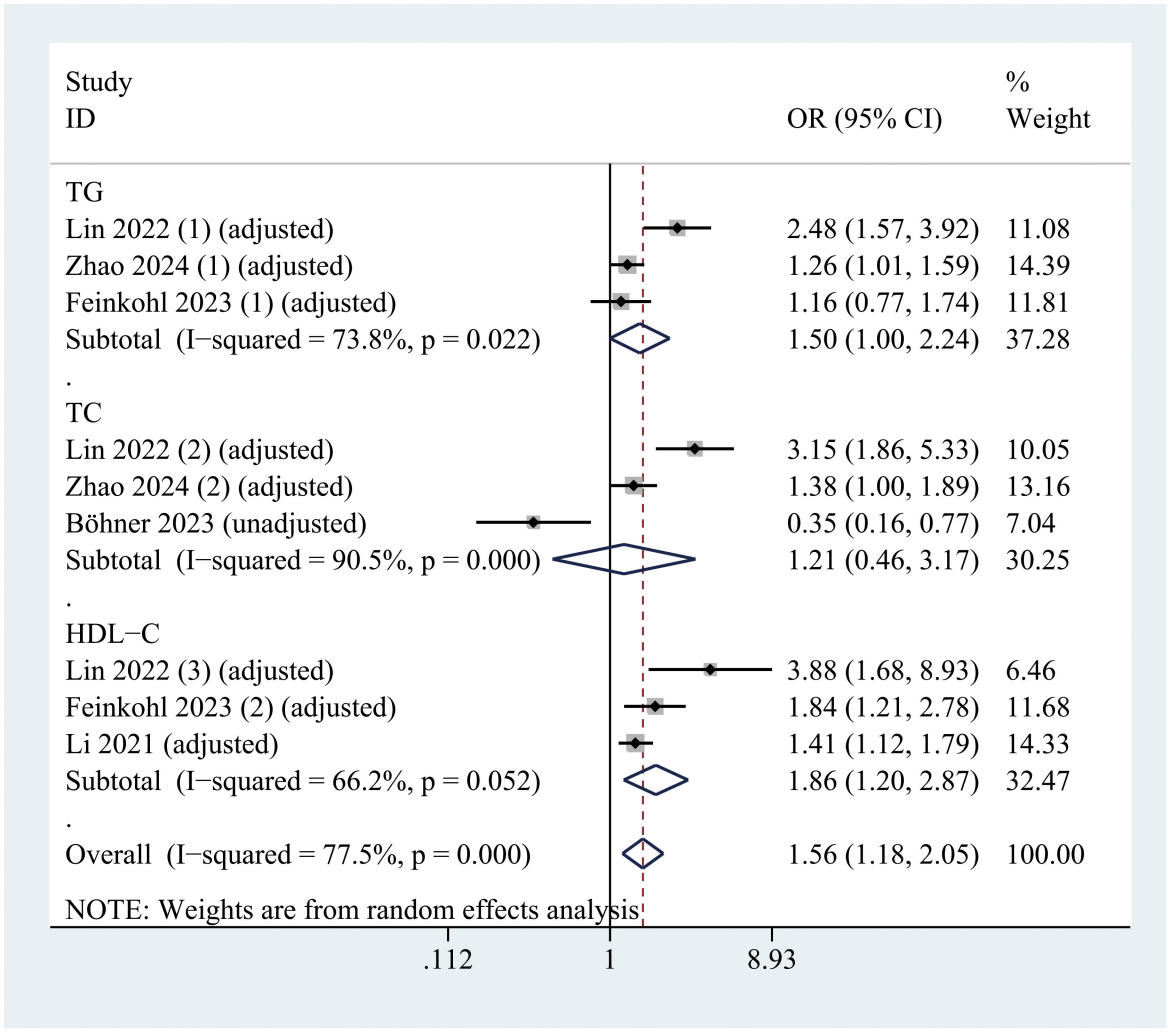
Forest plot illustrating the subgroup analysis evaluating the association between hyperlipidemia and risk of postoperative delirium, stratified by lipid profile classification.

In the subgroup analysis of hyperlipidemia classification, a reduced level of HDL-C (OR = 1.859; 95% CI 1.204–2.870; *P* = 0.005; *I*^2^ = 66.2%) was significantly associated with the occurrence of postoperative delirium (POD). However, no significant associations were observed between POD and TG levels (OR = 1.495; 95% CI 0.999–2.239; *P* = 0.051; *I*^2^ = 73.8%) or TC levels (OR = 1.208; 95% CI 0.460–3.173; *P* = 0.701; *I*^2^ = 90.5%) (Figure 4).

**Figure 5:**
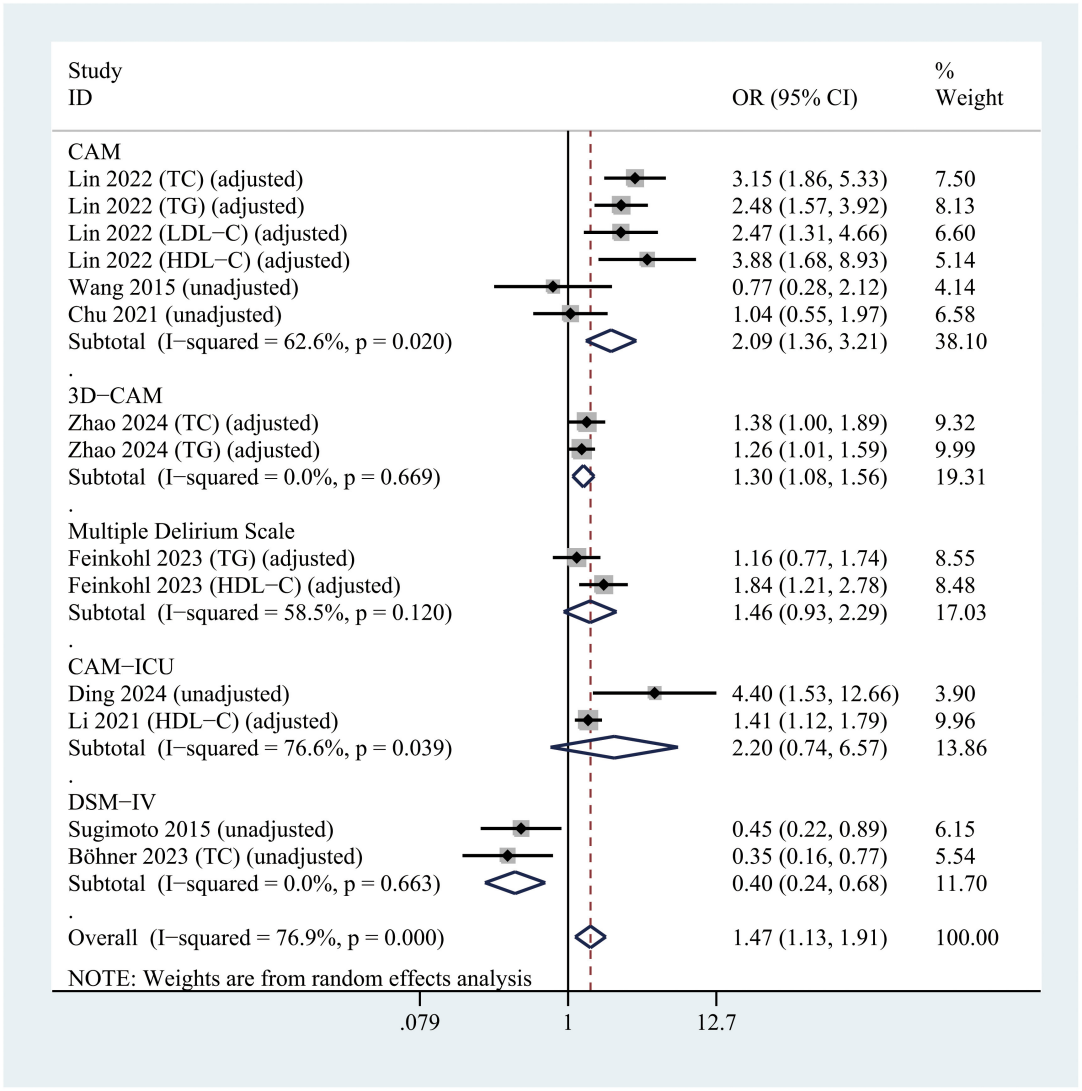
Forest plot of subgroup analysis assessing the relationship between hyperlipidemia and postoperative delirium risk, stratified by the Delirium assessment scale.

For delirium assessment, studies using the CAM (OR = 2.09; 95% CI 1.36–3.21; *P* = 0.001; *I*^2^ = 62.6%) and the 3D-CAM (OR = 1.30; 95% CI 1.08–1.56; *P* = 0.005; *I*^2^ = 0%) identified a significant association between hyperlipidemia and POD. In contrast, no significant association was found in studies using the CAM-ICU (OR = 2.20; 95% CI 0.74–6.57; *P* = 0.156; *I*^2^ = 76.6%) (Figure 5).

**Figure 6:**
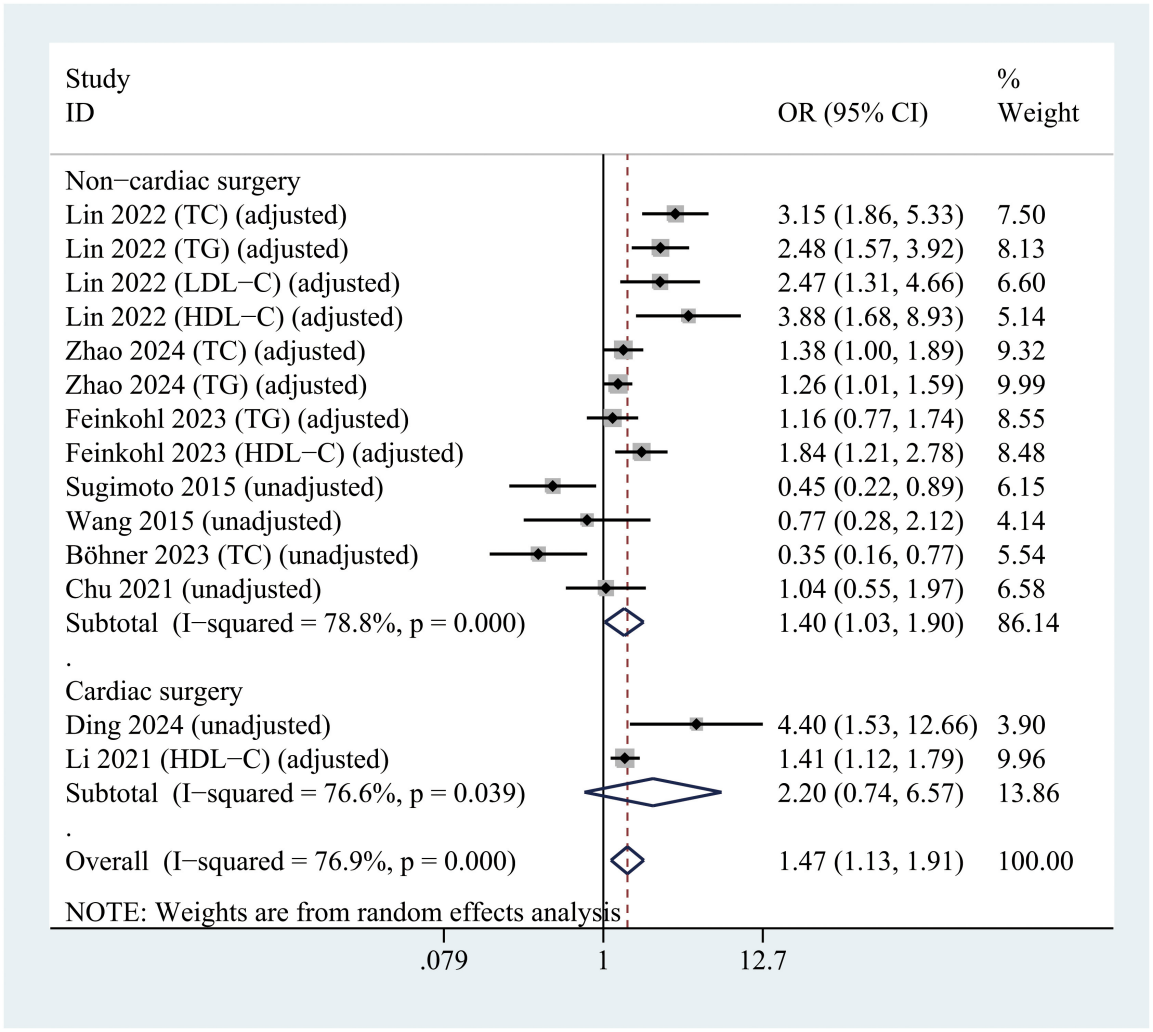
Forest plot of subgroup analysis assessing the relationship between hyperlipidemia and the risk of postoperative delirium based on the type of surgery.

A significant association between hyperlipidemia and POD was observed exclusively in patients undergoing non-cardiac surgery (OR = 1.4; 95% CI 1.03–1.90; *P* =0.033; *I*^2^ = 78.8%), whereas no such association was found in patients undergoing cardiac surgery (OR = 2.2; 95% CI 0.74–6.57; *P* = 0.156; *I*^2^ = 76.6%) (Figure 6).

In prospective cohort studies, hyperlipidemia was significantly associated with POD (OR = 1.67; 95% CI 1.23–2.28; *P* = 0.001; *I*^2^ = 77.0%). However, this association was not observed in retrospective case-control studies (OR = 0.92; 95% CI 0.48–1.77; *P* = 0.806; *I*^2^ = 79.6%) (Supplementary Figure 1).

Studies conducted in Asia revealed a significant association between hyperlipidemia and POD (OR = 1.62; 95% CI 1.22–2.17; *P* = 0.001; *I*^2^ = 75.6%), whereas studies in Europe did not demonstrate such an association (OR = 0.98; 95% CI 0.46–2.09; *P* = 0.956; *I*^2^ = 85.3%) (Supplementary Figure 2). Additionally, studies from China identified a significant correlation between hyperlipidemia and POD (OR = 1.79; 95% CI 1.37–2.32; *P* < 0.001; I^2^= 69.1%), in contrast to studies from other countries, which found no significant correlation (OR = 0.81; 95% CI 0.40–1.64; *P* =0.560; *I*^2^ = 69.1%) (Supplementary Figure 3).

The subgroup analysis based on variables adjusted revealed that hyperlipidemia was significantly associated with an increased risk of POD (OR = 1.75; 95% CI 1.40–2.20; *p* < 0.001; i^2^ = 67.5%) compared to patients without hyperlipidemia after adjusting for the multivariate factors; however, we found no significant correlation (OR = 0.85; 95% CI, 0.40–1.80; *p* = 0.664; I^2^ = 77.2%;) after adjusting for univariate factors (Supplementary Figure 4).

### Sensitivity analysis and publication bias

Sensitivity analysis revealed that no individual study significantly influenced the pooled odds ratio (OR) or its 95% confidence intervals (CIs) (Supplementary Figure 5). Exclusion of studies reporting only unadjusted ORs did not substantially alter the direction or significance of the pooled results (Supplementary Table 4). Furthermore, no significant publication bias was observed for the OR outcome. The funnel plot, which plotted the standard error of log OR against log OR, demonstrated symmetry. Both Egger's test (*P* = 0.795) and Begg's test (*P* = 0.913) confirmed the absence of statistically significant publication bias (Supplementary Figure 6).

## Discussion

This study is the first meta-analysis and systematic review to comprehensively evaluate the association between hyperlipidemia and postoperative delirium (POD). Our findings reveal that blood lipid levels were significantly higher in patients with POD compared to those without POD (NPOD). Moreover, patients with hyperlipidemia exhibited a significantly increased risk of developing POD relative to non-hyperlipidemic patients. However, the included studies exhibited heterogeneity in delirium diagnostic methods, hyperlipidemia classification, and study design (prospective vs. retrospective), which should be carefully considered when interpreting our findings.

Emerging evidence suggests that dyslipidemia may contribute to POD through multifaceted biological pathways. The association between hyperlipidemia and POD is likely mediated by neuroinflammation, oxidative stress, and cerebrovascular dysfunction. Elevated levels of low-density lipoprotein cholesterol (LDL-C) and triglyceride (TG) have been shown to promote the activation of microglia and astrocytes via the nuclear factor-kappa B (NF-κB) pathway, leading to increased production of pro-inflammatory cytokines (e.g., IL-6, TNF-α) in the central nervous system (CNS) (Yue et al., 2024). This neuroinflammatory cascade may disrupt synaptic plasticity and neurotransmitter balance (e.g., acetylcholine, dopamine), which are critical for maintaining cognitive function during surgical recovery (Liu et al., 2023). Furthermore, hypertriglyceridemia exacerbates oxidative stress by generating reactive oxygen species (ROS) through lipid peroxidation, potentially damaging neuronal mitochondria and impairing cerebral energy metabolism (Rojas-Gutierrez et al., 2017). In contrast, high-density lipoprotein cholesterol (HDL-C) plays a protective role through reverse cholesterol transport, reducing lipid accumulation and counteracting endothelial dysfunction, oxidative stress, inflammation, and thrombosis (Bates et al., 2017; Formiga et al., 2012, 2011). Studies consistently associate elevated serum HDL-C levels with enhanced cognitive function (Bates et al., 2017; Formiga et al., 2012, 2011). However, our meta-analysis revealed no significant association between hyperlipidemia (elevated TC/TG levels) and POD incidence. Notably, we identified a critical relationship between preoperative serum HDL-C levels and POD risk, suggesting that low preoperative HDL-C may serve as an independent risk factor for POD development.

Emerging evidence presents a complex interplay between dyslipidemia, lipid-lowering therapies, and postoperative delirium. While epidemiological studies frequently identify hyperlipidemia as a modifiable risk factor for POD, the therapeutic implications remain controversial. Preclinical models suggest that statins may attenuate neuroinflammation through pleiotropic effects on blood-brain barrier integrity and microglial activation, likely due to their pleiotropic effects beyond cholesterol reduction, including anti-inflammatory properties (via inhibition of isoprenoid synthesis) and endothelial stabilization (De Loecker and Preiser, 2012). However, randomized controlled trials (RCTs) have failed to consistently replicate these benefits, possibly due to heterogeneity in statin regimens (e.g., dosage, duration) and surgical context (e.g., cardiac vs. non-cardiac procedures) (Vallabhajosyula et al., 2017). Intriguingly, a lipid paradox has been observed: both severe hypertriglyceridemia and aggressive lipid-lowering correlate with increased POD incidence, suggesting a U-shaped relationship between lipid homeostasis and neurological outcomes (Chang et al., 2024). This phenomenon may reflect the dual role of cholesterol in neuroprotection (e.g., synaptic membrane integrity) vs. neurotoxicity (e.g., atherogenic plaque-induced ischemia) (Zhang and Liu, 2015).

The clinical implications of our findings highlight several key areas for improving POD risk management. Preoperative lipid profiling, including subtyping hyperlipidemia (e.g., distinguishing atherogenic dyslipidemia from isolated hypertriglyceridemia), may enhance POD risk stratification. The timing of lipid modulation is also critical, as chronic statin use appears beneficial, while abrupt perioperative withdrawal may trigger rebound inflammation (Pineda and Cubeddu, 2011). Additionally, novel therapeutic targets such as proprotein convertase subtilisin/kexin type 9 (PCSK9) inhibitors, which reduce LDL-C without crossing the blood-brain barrier, warrant further investigation for POD prevention (Agnello et al., 2024). Future research should focus on elucidating perioperative lipidomic changes and their interactions with blood-brain barrier permeability, alongside conducting large-scale RCTs to evaluate the efficacy of personalized lipid management protocols in high-risk surgical populations.

The potential sources of heterogeneity may include the following aspects. First, the methodologies for diagnosing delirium varied significantly across studies. Some studies employed the Confusion Assessment Method (CAM) (Feinkohl et al., 2023; Lin et al., 2022; Chu et al., 2021; Wang et al., 2015), a tool designed to assist non-psychiatric clinicians in identifying delirium, which has demonstrated high sensitivity and specificity consistent with the Diagnostic and Statistical Manual of Mental Disorders, Fourth Edition (DSM-IV) criteria. In contrast, other studies required delirium diagnoses to be made by psychiatrists using the Delirium Rating Scale (DRS) in conjunction with DSM-IV standards (Böhner et al., 2003). This variability in diagnostic tools may have introduced bias, as some tools are more sensitive to mild cases, while others focus on severe cases, potentially influencing the reported prevalence rates and associations. Additionally, the inconsistent and imprecise definitions of hyperlipidemia across studies may have further contributed to heterogeneity. While some studies provided explicit diagnostic criteria for hyperlipidemia (Zhao et al., 2024; Feinkohl et al., 2023; Li et al., 2021), others used more generalized definitions without distinguishing between low-density lipoprotein cholesterol (LDL-C) and high-density lipoprotein cholesterol (HDL-C). Such inconsistency could have led to the misclassification of participants as hyperlipidemic or non-hyperlipidemic, potentially overestimating or underestimating the true association between hyperlipidemia and outcomes, such as postoperative delirium. Furthermore, several studies were retrospective in design (Chu et al., 2021; Li et al., 2021; Sugimoto et al., 2015), potentially identifying only patients with pronounced symptoms that captured the attention of healthcare providers, thereby possibly missing cases of low-activity delirium. This limitation may lead to insufficient documentation of patients' mental states in medical records, resulting in an underrepresentation of delirium cases in retrospective analyses. In contrast, prospective studies can address this issue by implementing structured evaluation protocols that assess patients at least once daily. Variations in surgical techniques may also contribute to the discrepancies observed among studies. For instance, during cardiopulmonary bypass (CPB) in cardiac surgery, blood exposure to foreign materials can trigger a systemic inflammatory response, potentially leading to postoperative neurocognitive decline. Additionally, hemodynamic fluctuations during CPB may impair cerebral perfusion, causing cerebral ischemia—a well-established risk factor for POD (Hatami et al., 2022).

This study has several limitations. First, to minimize residual confounding bias, we prioritized the use of adjusted ORs in our analysis. However, differences in adjusted variables across studies may introduce bias, which represents a limitation of our study. Importantly, sensitivity analyses confirmed the robustness of our findings: excluding studies that reported only unadjusted ORs did not substantially change the direction or significance of the pooled results. Second, our study was characterized by substantial heterogeneity. Although we explored potential sources of heterogeneity through subgroup analyses, the limited number of studies with shared characteristics restricted the scope of these analyses. Finally, the variability in diagnostic tools and hyperlipidemia definitions across studies may have increased heterogeneity and bias, ultimately limiting the generalizability of our findings.

## Conclusions

Blood lipid levels were significantly elevated in POD patients compared to NPOD patients. Hyperlipidemia was significantly associated with an increased risk of POD, highlighting its potential role as a risk factor.

## Data Availability

The original contributions presented in the study are included in the article/Supplementary material, further inquiries can be directed to the corresponding author.

## Supplementary material

Supplementary Figure 1 Forest plot of subgroup analysis assessing the relationship between hyperlipidemia and the risk of postoperative delirium, stratified by the research types.

Supplementary Figure 2 Forest plot presenting the subgroup analysis of the association between hyperlipidemia and postoperative delirium risk, stratified by geographical region.

Supplementary Figure 3 Forest plot of subgroup analysis evaluating the association between hyperlipidemia and the risk of postoperative delirium, stratified by country.

Supplementary Figure 4 Forest plot illustrating the subgroup analysis evaluating the association between hyperlipidemia and postoperative delirium risk, stratified by the extent of covariate adjustment.

Supplementary Figure 5 Sensitivity analyses of OR data.

Supplementary Figure 6 Funnel plot of the standard error of log OR by log OR.

Supplementary File 1 The Search strategy for pubmed.

Supplementary File 2 PRISMA checklist.

Supplementary Table 1 Characteristics of the included studies.

Supplementary Table 2 Newcastle-Ottawa Scale criteria for case-control studies.

Supplementary Table 3 Newcastle-Ottawa Scale criteria for quality of cohort studies.

Supplementary Table 4 Sensitivity Analysis by Excluding Studies with Only Unadjusted Odds Ratios (ORs).
