# Supplementary figures and images for: Association between hyperlipidemia and postoperative delirium risk: a systematic review and meta-analysis
Source: Front Aging Neurosci. 2025 Mar 18;17:1544838. doi: 10.3389/fnagi.2025.1544838 (PMC11959067; doi:10.3389/fnagi.2025.1544838)

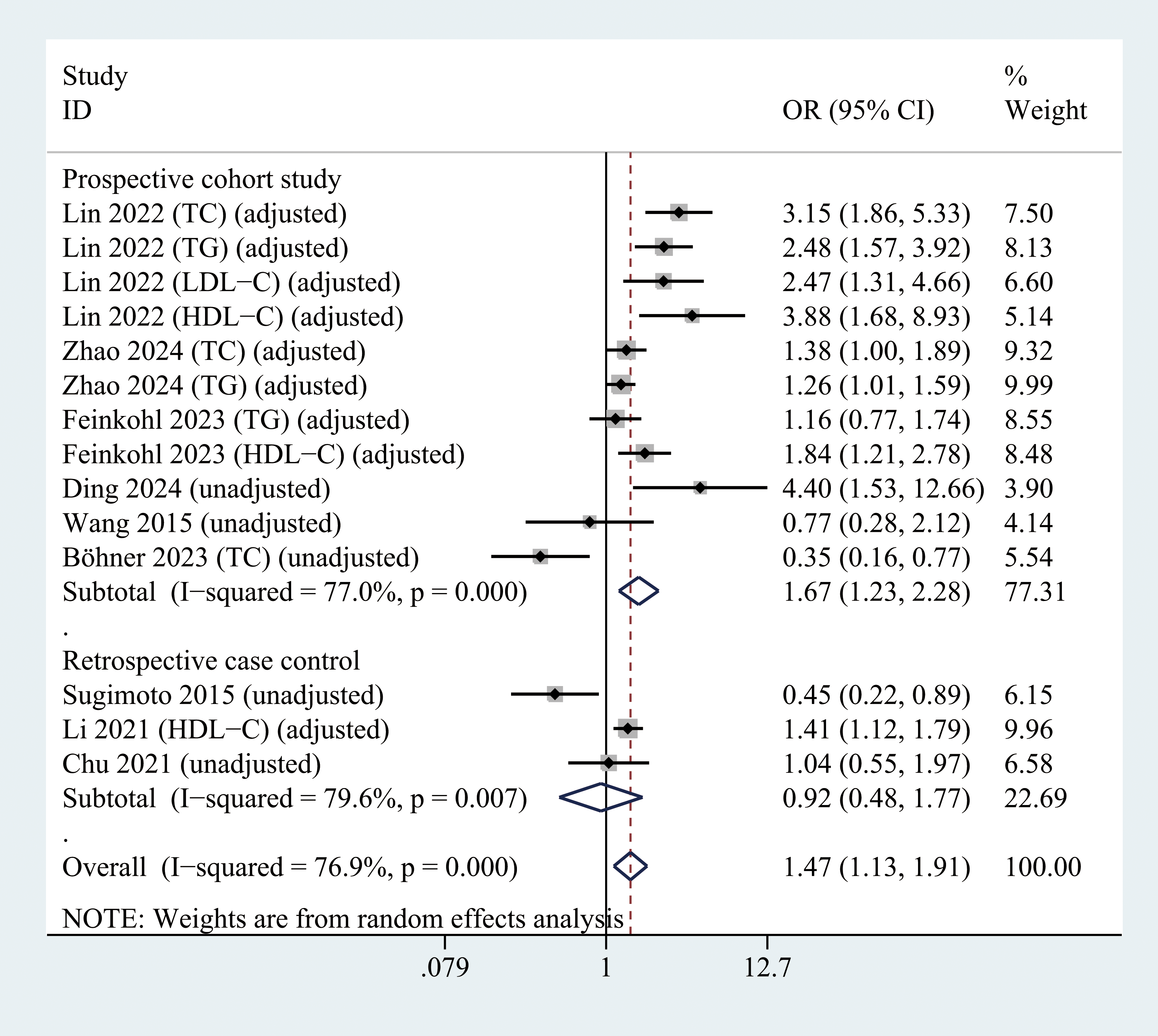

Supplement: Supplementary Figure 1 — Forest plot of subgroup analysis assessing the relationship between hyperlipidemia and the risk of postoperative delirium, stratified by the research types. [file Image_1.tif]

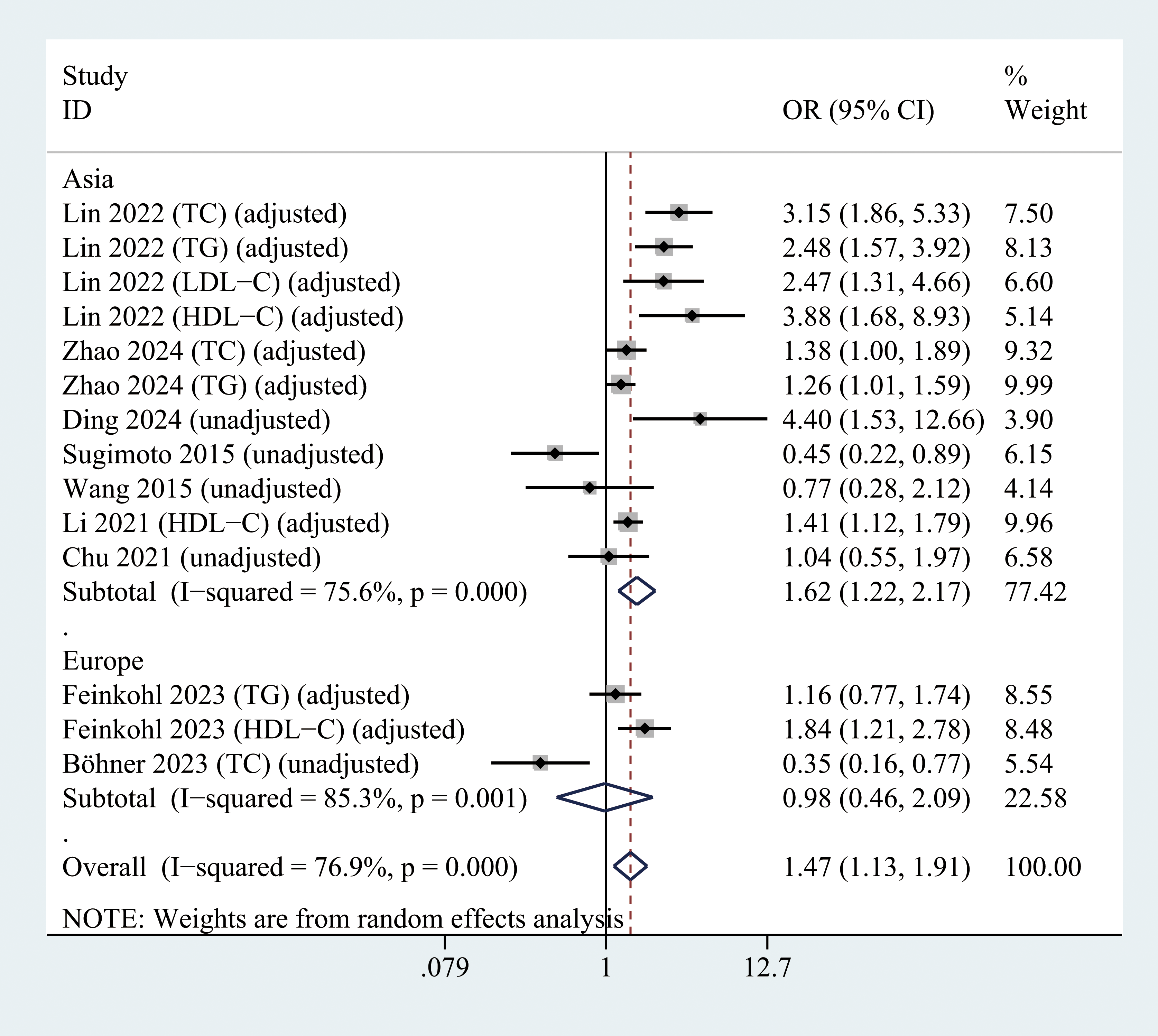

Supplement: Supplementary Figure 2 — Forest plot presenting the subgroup analysis of the association between hyperlipidemia and postoperative delirium risk, stratified by geographical region. [file Image_2.tif]

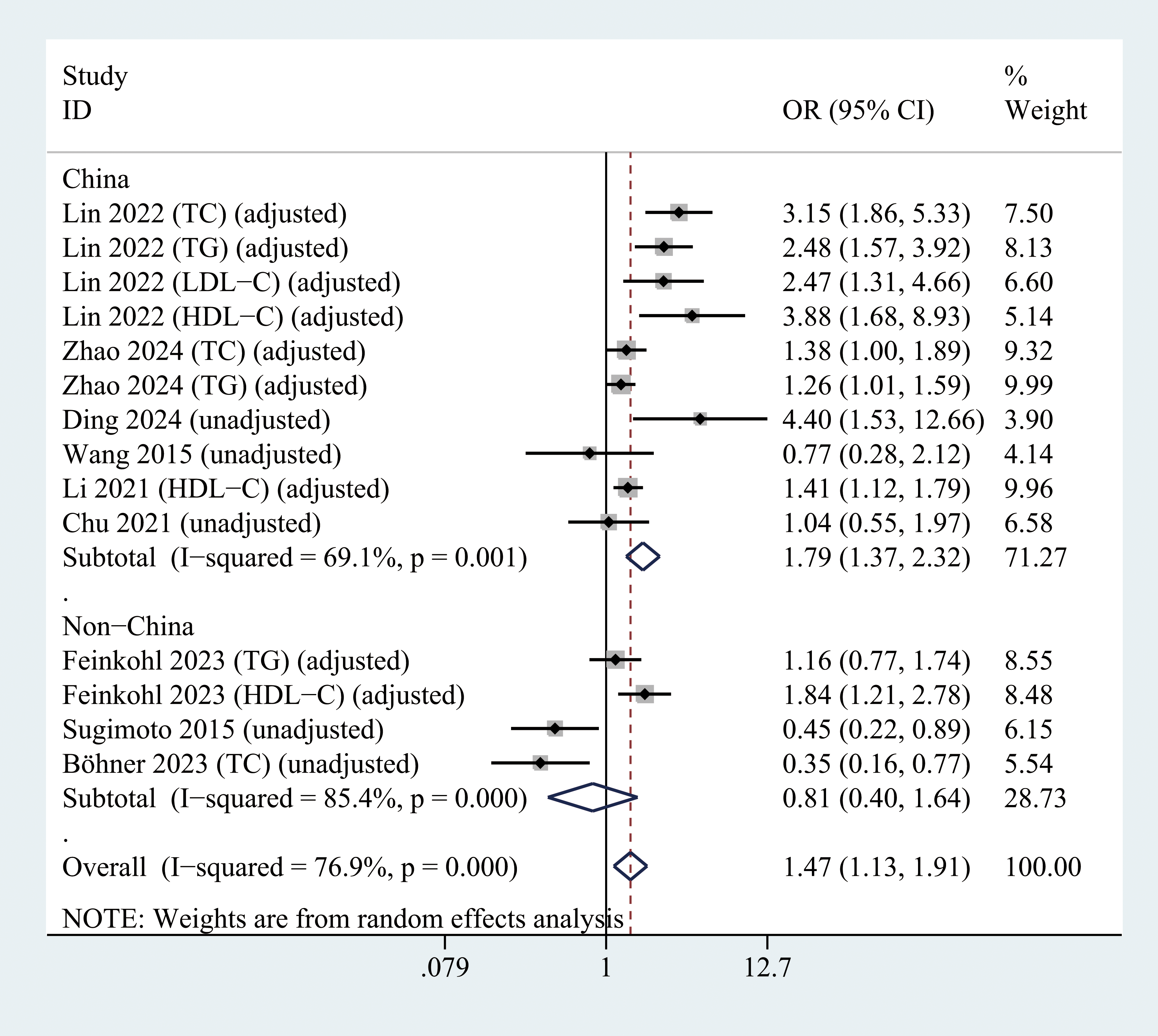

Supplement: Supplementary Figure 3 — Forest plot of subgroup analysis evaluating the association between hyperlipidemia and the risk of postoperative delirium, stratified by country. [file Image_3.tif]

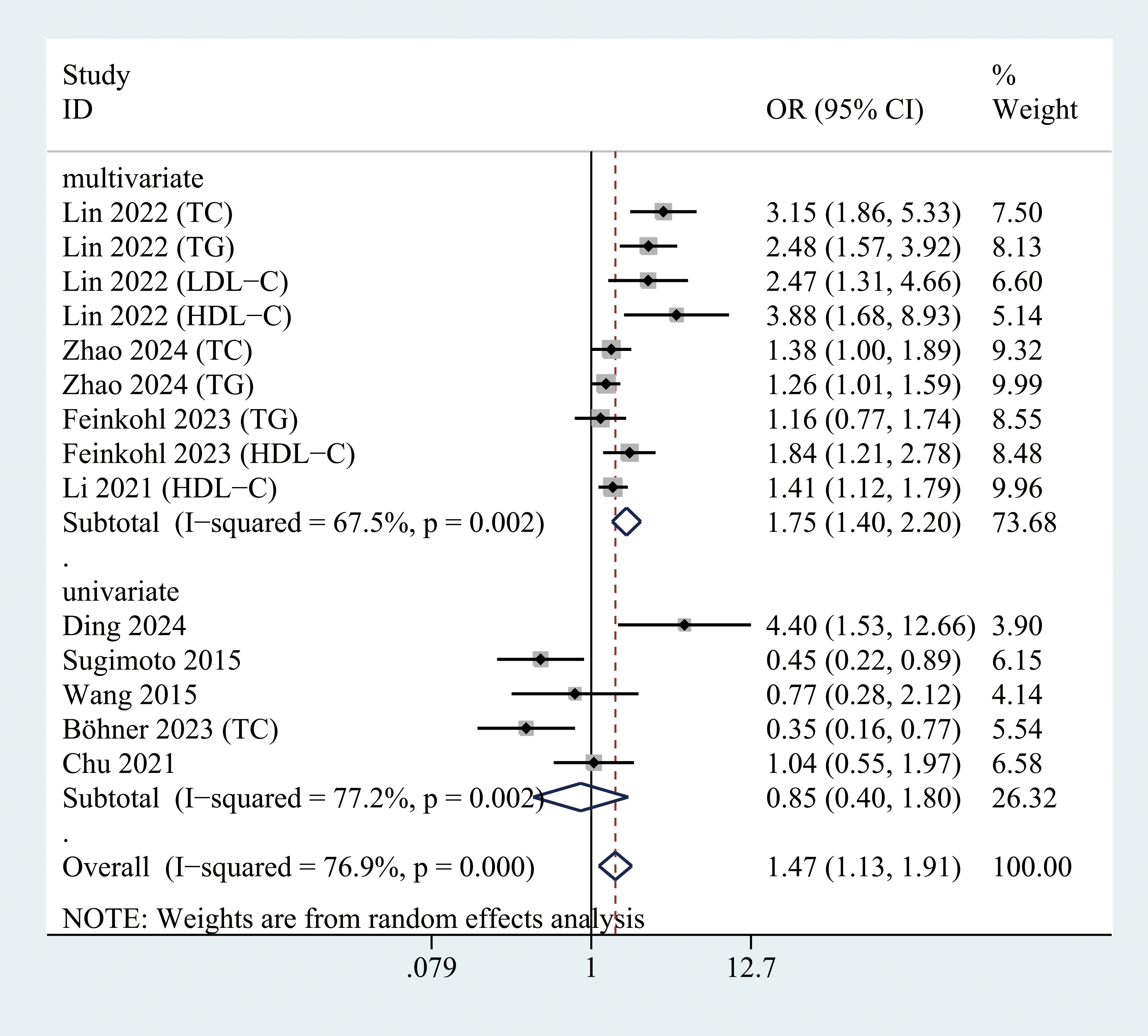

Supplement: Supplementary Figure 4 — Forest plot illustrating the subgroup analysis evaluating the association between hyperlipidemia and postoperative delirium risk, stratified by the extent of covariate adjustment. [file Image_4.tif]

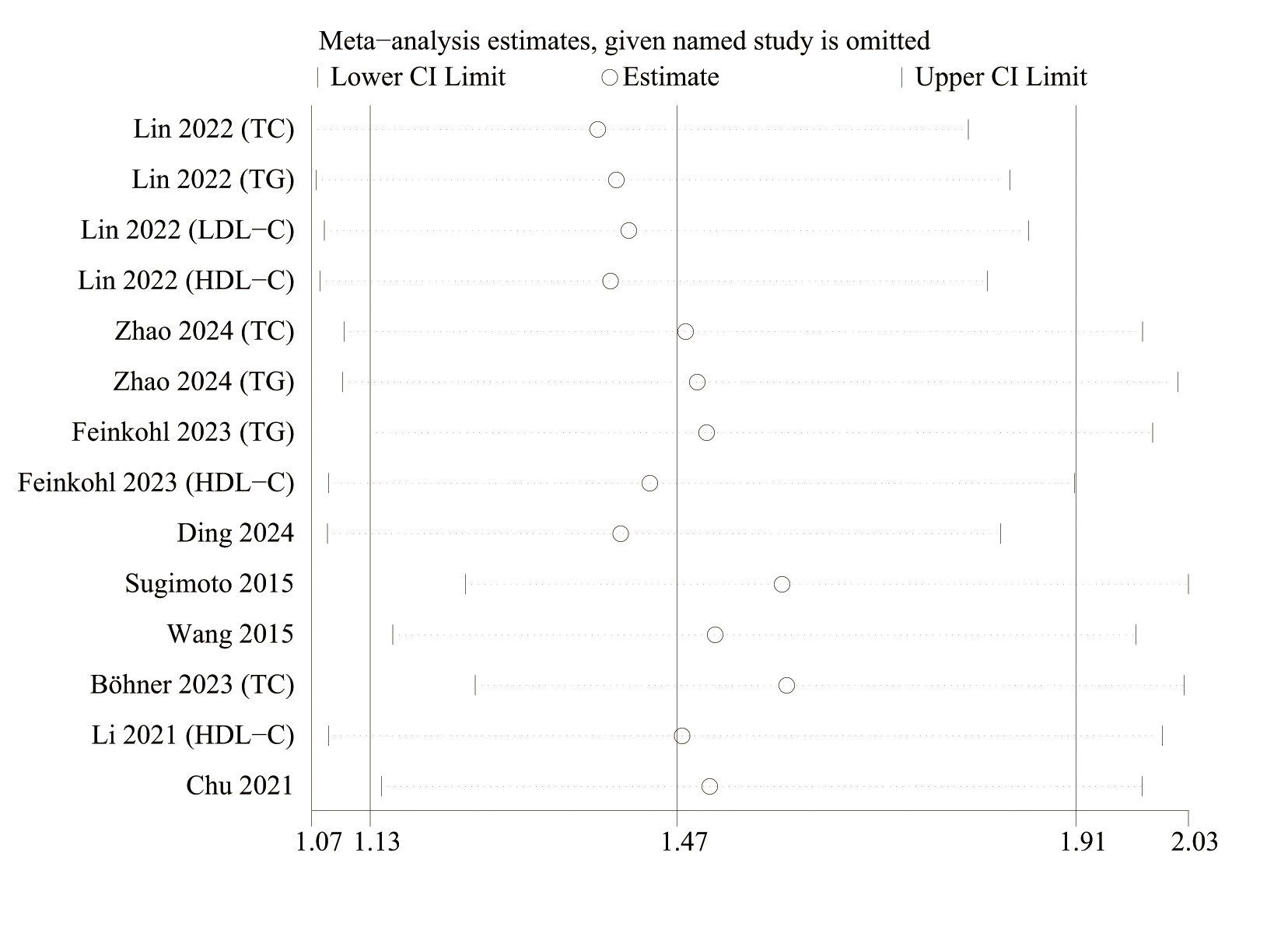

Supplement: Supplementary Figure 5 — Sensitivity analyses of OR data. [file Image_5.tif]

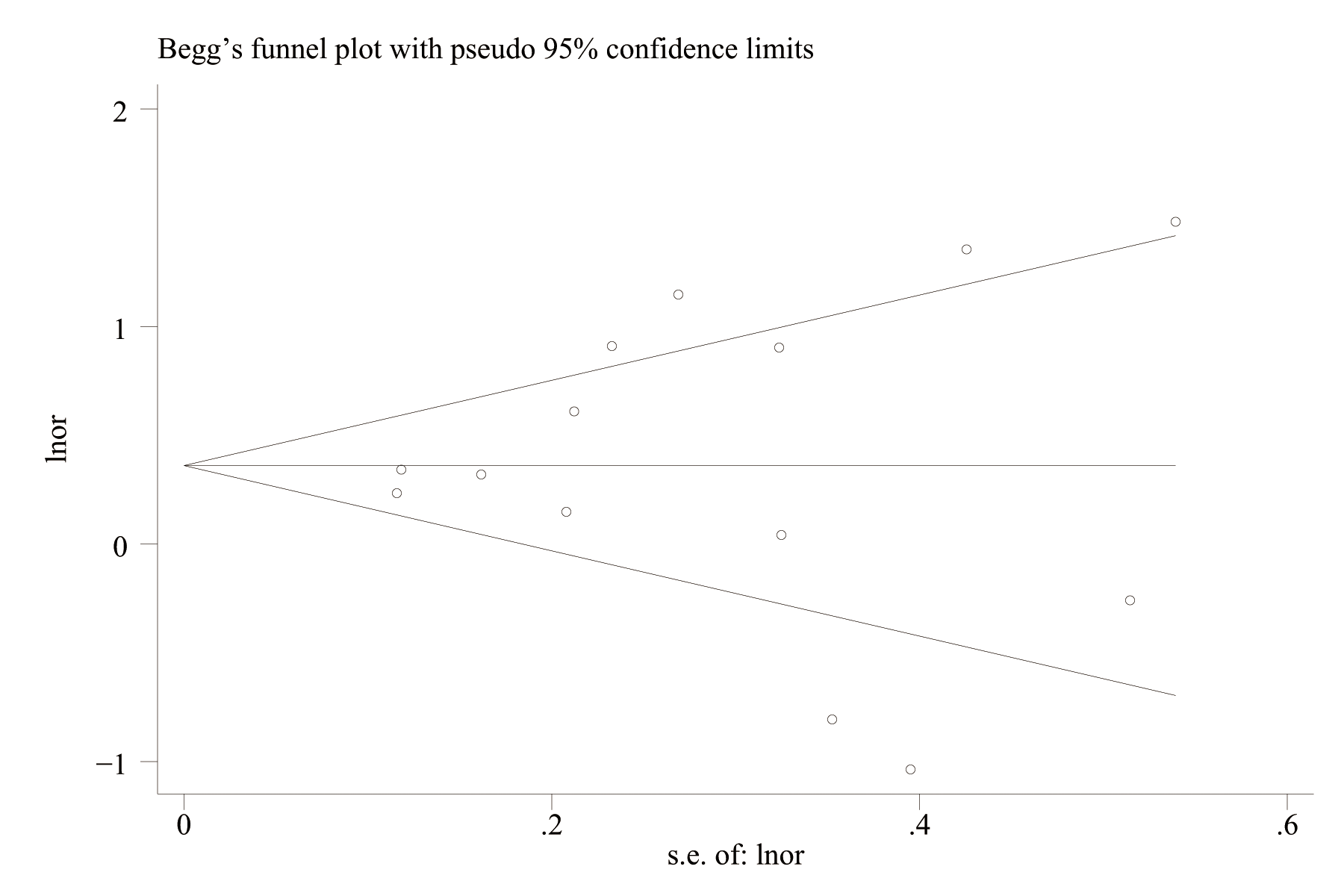

Supplement: Supplementary Figure 6 — Funnel plot of the standard error of log OR by log OR. [file Image_6.tif]
