# Supplementary material for: Association between hyperlipidemia and postoperative delirium risk: a systematic review and meta-analysis
Source: Front Aging Neurosci. 2025 Mar 18;17:1544838. doi: 10.3389/fnagi.2025.1544838 (PMC11959067; doi:10.3389/fnagi.2025.1544838)
Supplement: Supplementary File 1 — The Search strategy for pubmed. [file Table_1.docx]

**Search strategy in pubmed**

("Delirium"[MeSH Terms] OR ("Delirium"[MeSH Terms] OR "Delirium"[All Fields] OR ("Delirium"[All Fields] AND "mixed"[All Fields] AND "origin"[All Fields]) OR "delirium of mixed origin"[All Fields]) OR ("Delirium"[MeSH Terms] OR "Delirium"[All Fields] OR ("mixed"[All Fields] AND "origin"[All Fields] AND "Delirium"[All Fields])) OR ("Delirium"[MeSH Terms] OR "Delirium"[All Fields] OR ("mixed"[All Fields] AND "origin"[All Fields] AND "deliriums"[All Fields])) OR ("Delirium"[MeSH Terms] OR "Delirium"[All Fields] OR ("subacute"[All Fields] AND "Delirium"[All Fields]) OR "subacute delirium"[All Fields]) OR ("Delirium"[MeSH Terms] OR "Delirium"[All Fields] OR ("deliriums"[All Fields] AND "subacute"[All Fields])) OR ("Delirium"[MeSH Terms] OR "Delirium"[All Fields] OR ("Delirium"[All Fields] AND "subacute"[All Fields])) OR ("Delirium"[MeSH Terms] OR "Delirium"[All Fields] OR ("subacute"[All Fields] AND "deliriums"[All Fields]))) AND ("hyperlipidemias"[MeSH Terms] OR ("hyperlipidaemia"[All Fields] OR "hyperlipidemias"[MeSH Terms] OR "hyperlipidemias"[All Fields] OR "hyperlipidemia"[All Fields] OR "hyperlipidaemias"[All Fields]) OR ("hyperlipidemias"[MeSH Terms] OR "hyperlipidemias"[All Fields] OR "hyperlipemia"[All Fields] OR "hyperlipemias"[All Fields]) OR ("hyperlipidemias"[MeSH Terms] OR "hyperlipidemias"[All Fields] OR "hyperlipemia"[All Fields] OR "hyperlipemias"[All Fields]) OR ("hyperlipidemias"[MeSH Terms] OR "hyperlipidemias"[All Fields] OR "lipidaemia"[All Fields] OR "lipidemia"[All Fields]) OR ("hyperlipidemias"[MeSH Terms] OR "hyperlipidemias"[All Fields] OR "lipidemias"[All Fields]) OR ("hyperlipidemias"[MeSH Terms] OR "hyperlipidemias"[All Fields] OR "lipaemia"[All Fields] OR "lipemia"[All Fields]) OR ("hyperlipidemias"[MeSH Terms] OR "hyperlipidemias"[All Fields] OR "lipemias"[All Fields]) OR ("hypertriglyceridemia"[MeSH Terms] OR ("hypertriglyceridaemia"[All Fields] OR "hypertriglyceridemia"[MeSH Terms] OR "hypertriglyceridemia"[All Fields] OR "hypertriglyceridaemias"[All Fields] OR "hypertriglyceridemias"[All Fields])) OR ("hypercholesterolemia"[MeSH Terms] OR ("hypercholesterolaemia"[All Fields] OR "hypercholesterolemia"[MeSH Terms] OR "hypercholesterolemia"[All Fields] OR "hypercholesterolaemias"[All Fields] OR "hypercholesterolemias"[All Fields]) OR ("hypercholesterolemia"[MeSH Terms] OR "hypercholesterolemia"[All Fields] OR "hypercholesteremia"[All Fields]) OR ("hypercholesterolemia"[MeSH Terms] OR "hypercholesterolemia"[All Fields] OR "hypercholesteremias"[All Fields]) OR ("hypercholesterolemia"[MeSH Terms] OR "hypercholesterolemia"[All Fields] OR ("elevated"[All Fields] AND "cholesterol"[All Fields]) OR "elevated cholesterol"[All Fields]) OR ("hypercholesterolemia"[MeSH Terms] OR "hypercholesterolemia"[All Fields] OR ("cholesterol"[All Fields] AND "elevated"[All Fields]) OR "cholesterol elevated"[All Fields]) OR ("hypercholesterolemia"[MeSH Terms] OR "hypercholesterolemia"[All Fields] OR ("cholesterols"[All Fields] AND "elevated"[All Fields])) OR ("hypercholesterolemia"[MeSH Terms] OR "hypercholesterolemia"[All Fields] OR ("elevated"[All Fields] AND "cholesterols"[All Fields]) OR "elevated cholesterols"[All Fields]) OR ("hypercholesterolemia"[MeSH Terms] OR "hypercholesterolemia"[All Fields] OR ("high"[All Fields] AND "cholesterol"[All Fields] AND "levels"[All Fields]) OR "high cholesterol levels"[All Fields]) OR ("hypercholesterolemia"[MeSH Terms] OR "hypercholesterolemia"[All Fields] OR ("cholesterol"[All Fields] AND "level"[All Fields] AND "high"[All Fields]) OR "cholesterol level high"[All Fields]) OR ("hypercholesterolemia"[MeSH Terms] OR "hypercholesterolemia"[All Fields] OR ("cholesterol"[All Fields] AND "levels"[All Fields] AND "high"[All Fields]) OR "cholesterol levels high"[All Fields]) OR ("hypercholesterolemia"[MeSH Terms] OR "hypercholesterolemia"[All Fields] OR ("high"[All Fields] AND "cholesterol"[All Fields] AND "level"[All Fields]) OR "high cholesterol level"[All Fields]) OR ("hypercholesterolemia"[MeSH Terms] OR "hypercholesterolemia"[All Fields] OR ("level"[All Fields] AND "high"[All Fields] AND "cholesterol"[All Fields]) OR "level high cholesterol"[All Fields]) OR ("hypercholesterolemia"[MeSH Terms] OR "hypercholesterolemia"[All Fields] OR ("levels"[All Fields] AND "high"[All Fields] AND "cholesterol"[All Fields]) OR "levels high cholesterol"[All Fields])))
