# Supplementary material for: Association between hyperlipidemia and postoperative delirium risk: a systematic review and meta-analysis
Source: Front Aging Neurosci. 2025 Mar 18;17:1544838. doi: 10.3389/fnagi.2025.1544838 (PMC11959067; doi:10.3389/fnagi.2025.1544838)
Supplement: Supplementary Table 1 — Characteristics of the included studies. [file Table_2.docx]

**Supplement Table 1 Characteristics of the included studies**

| **Author(year)** | **Country** | **Type of Surgery** | **Type of Study** | **Number of patients** | **Delirium Assessment Scale** | **POD evaluation time** | **Type of anesthesia** | **Definition of hyperlipidemia** | **Variables in the multivariate model** | **Number of postoperative delirium** | **Number of Non-postoperative delirium** |
| --- | --- | --- | --- | --- | --- | --- | --- | --- | --- | --- | --- |
| Ding 2024 | China | On-pump cardiac surgery | Prospective cohort study | 130 | CAM-ICU | NA | Intravenous inhalation combined anesthesia | NA | Univariate | 32 | 98 |
| Zhao 2024 | China | Colorectal cancer surgery | Prospective cohort study | 555 | 3D-CAM | Three days postoperatively | Intravenous inhalation combined anesthesia | TG ≥ 1.7 mmol/L or TC ≥ 5.2 mmol/L or LDL-C ≥ 3.4 mmol/L or HDL-C <1.0mmol/L | Multivariate: education level, TG, TC, dietary habits, hypertension, SAS, and postoperative TMAO | 100 | 455 |
| Feinkohl 2023 | Germany | Various surgeries | Prospective cohort study | 765 | NDSS/CAM/CAM-ICU/Medical Record Description | One week postoperatively | NA | TG ≥ 1.9 mmol/L or HDL-C<1.0mmol/L | Multivariate: age, sex, analysis laboratory, analysis batch, surgery type, coronary heart disease, transient ischaemic attack, stroke, depression, and anesthesia duration | 149 | 616 |
| Lin 2022 | China | Hip and knee replacement surgery | Prospective cohort study | 562 | CAM | One week postoperatively | Epidural anesthesia | NA | Multivariate: age, sex, education, and MMSE score | 66 | 496 |
| Sugimoto 2015 | Japan | Open abdominal aortic aneurysm repair | Retrospective case control study | 397 | DSM-IV | During hospitalization | General anesthesia | NA | Univariate | 46 | 351 |
| Wang 2015 | China | Orthopedic surgery | Prospective cohort study | 200 | CAM | One week postoperatively | General anesthesia + epidural anesthesia | NA | Univariate | 17 | 183 |
| Böhner 2003 | Germany | Vascular surgery | Prospective cohort study | 153 | DSM IV+DRS | One week postoperatively | General anesthesia | TG ≥ 1.7 mmol/L | Univariate | 60 | 93 |
| Li 2021 | China | Coronary artery bypass grafting surgery | Retrospective case control study | 1462 | CAM-ICU | During ICU period | General anesthesia | HDL<1.0mmol/L | Multivariate: age, drinking, diabetes, strokes, ICA stenosis, HDL-C, EF, CPB, IABP, Cr, PO2, LCOS, pneumonia, MODS, et al. | 560 | 902 |
| Chu 2021 | China | Hip fracture surgery | Retrospective case control study | 462 | CAM | During hospitalization | NA | NA | Univariate | 74 | 388 |

NA：Not available. CAM = Confusion Assessment Method; TMAO = Trimethylamine N-oxide; CAM-ICU = Confusion Assessment Method for Intensive Care Unit; 3D-CAM = 3-Minute Diagnostic Interview for Confusion Assessment Method Chinese version; NDSS = Nursing Delirium Screening Scale; MMSE = The Mini-Mental State Examination; DSM-IV = Diagnostic and Statistical Manual of Mental Disorders 4th Edition; TC = Total cholesterol; TG = Triglycerides; SAS = SAS, Sedation-agitation scale; DRS = Delirium Rating Scale; ICA = Internal carotid artery; HDL-C = High-density lipoprotein cholesterol; EF = Ejection fraction; CPB = Cardiopulmonary bypass; IABP = Intra-aortic balloon pump; Cr = Creatinine; PO2 = Arterial oxygen partial; LCOS = Low cardiac output syndrome; MODS = Multiple organ dysfunction syndrome

**Supplement table 2 Newcastle-Ottawa Scale criteria for case-control studies**

| **Author（year）** | **Is the case definition adequate?** | **Representativeness of the cases** | **Selection of controls** | **Definition of controls** | **Comparability of cases and controls on the basis of the design or analysis** | **Ascertainment of exposure** | **Same method of ascertainment for cases and controls** | **Non-response rate** | **Total scores** |
| --- | --- | --- | --- | --- | --- | --- | --- | --- | --- |
| **Chu 2021** | * | * | – | * | * | * | * | – | 6 |
| **Li 2021** | * | * | – | * | * | * | * | – | 6 |
| **Sugimoto 2015** | * | * | – | * | * | * | * | – | 6 |

A single asterisk (*) indicates that one point was awarded.

**Supplement table 3 Newcastle-Ottawa Scale criteria for quality of cohort studies**

| **Author and Year** | **Selection** | | | | **Comparability** | **Outcome** | | | **Total scores** |
| --- | --- | --- | --- | --- | --- | --- | --- | --- | --- |
|  | **Representativeness** | **Selection** | **Ascertainment** | **Outcome** | **Comparability** | **Assessment** | **Follow-up** | **Adequacy of follow-up** |  |
| **Ding 2024** | * | * | * | * | * | * | 0 | * | 7 |
| **Zhao 2024** | * | * | * | * | ** | * | * | * | 9 |
| **Feinkohl 2023** | * | * | * | * | * | * | * | * | 8 |
| **Lin 2022** | * | * | * | * | ** | * | * | * | 9 |
| **Wang 2015** | 0 | * | * | * | * | * | * | * | 7 |
| **Böhner 2003** | * | * | * | * | * | * | * | * | 8 |

A single asterisk (*) indicates that one point was awarded.

**Supplement table 4. Sensitivity Analysis by Excluding Studies with Only Unadjusted Odds Ratios (ORs)**

| **Category** | **Number of Trails** | **OR and 95%CI** | **I^2^(%)** | **P** |
| --- | --- | --- | --- | --- |
| Postoperative delirium |  |  |  |  |
| All studies | 9 | 1.47 (1.13, 1.91) | 76.9 | 0.004 |
| Excluding studies with unadjusted ORs | 4 | 1.75 (1.40, 2.20) | 67.5 | <0.001 |
| Triglyceride (TG) |  |  |  |  |
| All studies | 3 | 1.21 (0.46, 3.17) | 90.5 | 0.701 |
| Excluding studies with only unadjusted ORs | 2 | 2.02 (0.90, 4.55) | 85.6 | 0.088 |
| Confusion Assessment Method (CAM) scale |  |  |  |  |
| All studies | 6 | 2.09 (1.36, 3.21) | 62.6 | 0.001 |
| Excluding studies with unadjusted ORs | 4 | 2.80 (2.11, 3.72) | 0 | <0.001 |
| Non-cardiac surgery |  |  |  |  |
| All studies | 8 | 1.40 (1.03, 1.90) | 78.8 | 0.033 |
| Excluding studies with unadjusted ORs | 4 | 1.85 (1.41, 2.45) | 70.7 | <0.001 |
| Prospective cohort study |  |  |  |  |
| All studies | 6 | 1.67 (1.23, 2.28) | 77.0 | 0.001 |
| Excluding studies with unadjusted ORs | 3 | 1.85 (1.41, 2.44) | 70.7 | <0.001 |
| Asia |  |  |  |  |
| All studies | 8 | 1.62 (1.22, 2.17) | 75.6 | 0.001 |
| Excluding studies with unadjusted ORs | 4 | 1.88 (1.42, 2.48) | 72.8 | <0.001 |
| Europe |  |  |  |  |
| All studies | 3 | 0.98 (0.46, 2.09) | 85.3 | 0.956 |
| Excluding studies with unadjusted ORs | 2 | 1.46 (0.93, 2.29) | 58.5 | 0.102 |
| China |  |  |  |  |
| All studies | 7 | 1.79 (1.37, 2.32) | 69.1 | <0.001 |
| Excluding studies with unadjusted ORs | 4 | 1.88 (1.42, 2.48) | 72.8 | <0.001 |
| Non-China |  |  |  |  |
| All studies | 4 | 0.81 (0.40, 1.64) | 85.4 | 0.560 |
| Excluding studies with unadjusted ORs | 2 | 1.46 (0.93, 2.29) | 58.5 | 0.102 |
